# Determinants of Oxygen and Carbon Dioxide Transfer during Extracorporeal Membrane Oxygenation in an Experimental Model of Multiple Organ Dysfunction Syndrome

**DOI:** 10.1371/journal.pone.0054954

**Published:** 2013-01-29

**Authors:** Marcelo Park, Eduardo Leite Vieira Costa, Alexandre Toledo Maciel, Débora Prudêncio e Silva, Natalia Friedrich, Edzangela Vasconcelos Santos Barbosa, Adriana Sayuri Hirota, Guilherme Schettino, Luciano Cesar Pontes Azevedo

**Affiliations:** 1 Research and Education Institute, Hospital Sírio-Libanês, São Paulo, Brazil; 2 Intensive Care Unit, Hospital das Clinicas, University of São Paulo School of Medicine, São Paulo, Brazil; The Ohio State Unversity, United States of America

## Abstract

Extracorporeal membrane oxygenation (ECMO) has gained renewed interest in the treatment of respiratory failure since the advent of the modern polymethylpentene membranes. Limited information exists, however, on the performance of these membranes in terms of gas transfers during multiple organ failure (MOF). We investigated determinants of oxygen and carbon dioxide transfer as well as biochemical alterations after the circulation of blood through the circuit in a pig model under ECMO support before and after induction of MOF. A predefined sequence of blood and sweep flows was tested before and after the induction of MOF with fecal peritonitis and saline lavage lung injury. In the multivariate analysis, oxygen transfer had a positive association with blood flow (slope = 66, P<0.001) and a negative association with pre-membrane PaCO_2_ (slope = −0.96, P = 0.001) and SatO_2_ (slope = −1.7, P<0.001). Carbon dioxide transfer had a positive association with blood flow (slope = 17, P<0.001), gas flow (slope = 33, P<0.001), pre-membrane PaCO_2_ (slope = 1.2, P<0.001) and a negative association with the hemoglobin (slope = −3.478, P = 0.042). We found an increase in pH in the baseline from 7.50[7.46,7.54] to 7.60[7.55,7.65] (P<0.001), and during the MOF from 7.19[6.92,7.32] to 7.41[7.13,7.5] (P<0.001). Likewise, the PCO_2_ fell in the baseline from 35 [32,39] to 25 [22,27] mmHg (P<0.001), and during the MOF from 59 [47,91] to 34 [28,45] mmHg (P<0.001). In conclusion, both oxygen and carbon dioxide transfers were significantly determined by blood flow. Oxygen transfer was modulated by the pre-membrane SatO_2_ and CO_2_, while carbon dioxide transfer was affected by the gas flow, pre-membrane CO_2_ and hemoglobin.

## Introduction

Extracorporeal membrane oxygenation (ECMO) has been successfully used to support patients with severe acute respiratory failure associated with refractory hypoxemia or uncompensated hypercapnia. [1]–[4] The choice of the ECMO settings depends on whether oxygenation or CO_2_ removal is the main indication of extracorporeal support. The concept that blood flow affects the transfer of oxygen and CO_2_, and sweep (gas) flow, that of CO_2_ [5] is supported by evidence in healthy animals using a spiral coiled rubber silicone membrane. [6] There is, however, limited information about the main determinants of oxygen and carbon dioxide transfer during ECMO using the modern polymethylpentene membrane in subjects with multiple organ failure (MOF). [7] Many clinical decisions at the bedside are based on accumulated experience or on simple physiological assumptions and have not been formally tested. For example, in one study, [1] hemoglobin was empirically maintained at 14 g/dL with the aim of optimizing oxygen transfer. Many other variables readily available at the bedside, such as body temperature, blood osmolality, and baseline blood gases could also alone or in combination influence ECMO efficiency.[6]; [7] The knowledge on which of these variables has the greatest impact on oxygenation and CO_2_ removal would be important to improve ECMO delivery.

During the passage of blood through the ECMO circuit, there are profound modifications of the partial pressure of oxygen (PO_2_) and of carbon dioxide (PCO_2_), and consequently pH. [7] These modifications affect the ionization of proteins [8] and their binding capacity [8]; [9] and cause electrolytes shifts between the intra and extra-cellular media or between intra and extra-vascular media. [10] These electrolytes alterations, although negligible in physiological conditions, may be unpredictable in non-physiological pH ranges. [9] Some of the ECMO-associated electrolyte disturbances can be clinically significant. For example, they can be associated with hemodynamic depression [11] and can affect the electrolyte clearance of renal replacement therapy when the blood is drained from the post-membrane ECMO circuit. [12] An adequate characterization of these post membrane alterations is of potential clinical importance and has not been carried out so far.

Based on basic physiological principles, we hypothesized that the oxygen transfer would be governed by the blood flow, hemoglobin concentration, blood temperature, and by pre-membrane oxygen saturation, while the CO_2_ transfer would be governed by temperature, gas flow, and by pH. Furthermore, we presumed that the passage of blood through the ECMO circuit would be associated with significant electrolyte disturbances. To test these hypotheses, we measured gas transfers as well as electrolyte concentrations pre and after membrane in animals supported with venous-venous ECMO, with assorted blood and sweep flows, before and after the induction of lung injury and sepsis. Our objective was to assess the determinants of oxygen and carbon dioxide transfer, and biochemical disturbances during the passage of blood through the polymethylpentene extracorporeal membrane applied to normal and MOF subjects.

## Methods

This study had approval from the Institutional Animal Research Ethics Committee from the Hospital Sírio Libanês in São Paulo – Brazil and was performed according to National Institutes of Health guidelines for the use of experimental animals. Instrumentation, surgical preparation, pulmonary injury, and induction of sepsis were performed as previously described. [13]–[15].

### Instrumentation and Surgical Preparation

The room temperature was set at 24° Celsius. Five domestic female Agroceres pigs (80 [79,81] kg) were anesthetized with thionembutal (10 mg/kg, Tiopental, Abbott, Brazil) and pancuronium bromide (0.1 mg/kg, Pavulon, AKZO Nobel, Brazil) and connected to a mechanical ventilator (Evita XL Dräger, Dräger, Luebeck, Germany) with the following parameters: tidal volume 8 mL/kg, end-expiratory pressure 5 cm H_2_O, FiO_2_ initially set at 100% and subsequently adjusted to maintain arterial saturation between 94–96%, and respiratory rate titrated to maintain PaCO_2_ between 35 and 45 mm Hg or an end-tidal CO_2_ (NICO, Dixtal Biomedica Ind. Com, Sao Paulo, Brazil) between 30 and 40 mmHg. The electrocardiogram, heart rate, oxygen saturation, and pressures of the animals were monitored with a multiparametric monitor (Infinity Delta XL, Dräger, Luebeck, Germany). Anesthesia was maintained during the study with midazolam (1–5 mg.kg^−1^.h^−1^) and fentanyl (5–10 mcg.kg^−1^.h^−1^) and muscular relaxation with pancuronium bromide (0.2 mg.kg^−1^.h^−1^). The adequate depth of anesthesia during the surgical period was evaluated with maintenance of physiological variables (heart rate and arterial pressure) and absence of reflexes (corneal and hind limb flexion response), as well as unresponsiveness to stimuli during manipulation. Supplementary boluses of 3–5 mcg/kg fentanyl and 0.1–0.5 mg/kg midazolam were administered as necessary.

The left external jugular vein was cannulated (guided by ultrasonography) to introduce a pulmonary artery catheter, and the right external jugular vein, to introduce a 25-cm ECMO return canulae (Edwards Lifesciences, Irvine, CA, USA). The right femoral vein was punctured for the insertion of a 55-cm ECMO drainage canulae (Edwards Lifesciences, Irvine, CA, USA), which was positioned close to the right atrium with the aid of trans-hepatic ultrasonographic visualization. Only the guidewires were inserted until the first baseline measurements after the stabilization period, when they were replaced by the ECMO catheters. After the insertion of the guidewires, an infusion of 1000 IU per hour of heparin was started. A central venous catheter and an invasive arterial blood pressure catheter were placed in the left femoral vein and artery, respectively.

Through a midline laparotomy, a cistostomy was performed, and a bladder catheter inserted. A 2-cm incision was performed in the descending colon, and 1.0 g/kg of fecal contents was removed and stored. The intestinal incision was sutured; two large-bore catheters were inserted in each flank of the animal, and the laparotomy was closed. During the surgical interventions, 15 mL.kg^−1^.h^−1^ of lactated Ringer’s were continuously infused, and boluses of 250 mL were used to maintain a systemic mean arterial blood pressure (ABPm) of 65 mmHg or greater, central venous pressure (CVP) of 8 mmHg or greater, and SvO_2_ greater than 65% until the end of instrumentation.

### Stabilization and Support of the Animals

After the end of the instrumentation, the animals were allowed to stabilize for 1 h. A continuous infusion of 3 mL.kg^−1^.h^−1^ of lactated Ringer’s was started at the beginning of the stabilization period and kept throughout the entire experiment. Blood glucose was monitored at least hourly. If the blood glucose was lower than 60 mg/dL, a solution of 40 mL of 50% glucose was infused.

When the animals became hypotensive (ABPm<65 mmHg), a bolus of 500 mL of lactated Ringer’s was infused. If the ABPm failed to rise above 65 mmHg after the bolus, an infusion of norepinephrine 0.1 mcg.kg^−1^.min^−1^ (Norepine, Opem Pharmaceuticals, São Paulo, Brazil) was started and titrated to an ABPm ≥ 65 and <80 mmHg.

### ECMO Priming, Starting, and Maintenance

The ECMO system (Permanent life support system - PLS, Jostra – Quadrox D, Maquet Cardiopulmonary, Hirrlingen, Germany) was primed with a 37° Celsius normal saline solution and connected to a centrifugal pump (Rotaflow, Jostra, Maquet Cardiopulmonary, Hirrlingen, Germany). With the circuit filled, 1000 IU of heparin were injected in the circulating fluid. The anticoagulation was monitored with the measurement of the activated coagulation time (ACT) at baseline and every 6 hours. The infusion of heparin was titrated to keep the ACT 1.5–2.5 times the first baseline ACT value. The CO_2_ partial pressure of the air exhaled from the respiratory membrane was measured with an EtCO_2_ probe Tonocap® (Datex -General Electric Healthcare, USA).

The PLS uses a polymethylpentene membrane; the tubes are coated with a bioactive and biopassive system (Bioline, Maquet Cardiopulmonary, Hirrlingen, Germany). [16] Two luer-locks were connected respectively in the pre and post membrane ports, in order to allow for the measurement of pressures and for the collection of blood samples. Pressure lines were connected to ports in the drainage tube (before the centrifugal pump), pre and post ECMO membrane; pressure measurements were performed in real time with a multiparametric monitor (Dx 2020, Dixtal Biomedica Ind. Com, Sao Paulo, Brazil).

During the extracorporeal circulation, if the system began to vibrate significantly or cavitate, or if the blood flow decreased despite unchanged rotations per minute in the pump, a standardized sequence of procedures was done: first, the position of the animal was gently modified with lateralization of the animal, and if necessary followed by a semi-recumbent positioning; second, the PEEP level was raised in 1 or 2 cmH_2_O. If the PEEP was higher than 6 cmH_2_O, a trial to lower the PEEP by 1 or 2 cmH_2_O could also be performed; third, boluses of 250 mL of lactated Ringer’s were tried.

### Measurements

The following data were collected: heart rate (HR), mean arterial blood pressure (ABPm), central venous pressure (CVP), mean pulmonary artery pressure (PAPm), pulmonary artery occluded pressure (PAOP), cardiac output (CO), core temperature, peripheral oxygen saturation, end-tidal CO_2_ (EtCO_2_), and mixed venous oxygen saturation (SvO_2_), PEEP, FiO_2_, auto-PEEP measured with 4 seconds of expiratory pause, plateau pressure with 2 seconds of inspiratory pause, and peak pressure. Blood samples from the pulmonary and femoral arteries were collected and analyzed in a standard radiometer ABL 600 (Radiometer, Copenhagen, Denmark). The sample from the femoral artery was used for the biochemical analysis.

We collected the baseline data after 30 min of ECMO at a blood flow of 1.5 L/min and with the gas (sweep) flow turned off, i.e. without gas exchange. The formulas for calculations of respiratory, hemodynamic, and ECMO parameters are available in the electronic supplement that accompanies this manuscript. The standard formulas used to the calculus [14]; [15]; [17]; [18] are shown in the Text S1– Online supplement.

### Varying Blood and Sweep Flow

A sequence of blood and sweep flows was chosen based on the usual needs at bedside. In this way, a blood flow of 1500 and 3000 mL are commonly used in ECMO support, and a blood flow of 500 mL can be reached during interventional lung assist support. The sweep flows were chosen in order to allow measurements of gas transfer in a blood/sweep flow ratio of 2∶1, 1∶1 and 1∶2.

After the baseline measurements, the blood flow was set to 5000 mL/min and the sweep flow, to 5.0 L/min. After 10 minutes, the blood flow was reduced to 1500 mL/min, and the sweep flow was set at two-minute intervals to 1.5, 1.0, and 3.0 L/min. The blood flow was then increased to 3000 mL/min, and the sweep flow was set to 1.5, 3.0, and 6.0 L/min in two-minute steps. Finally, the blood flow was reduced to 500 mL/min and the sweep flow was set using 0.25, 1.0, and 0.5 L/min at two-minute intervals. At the end of each sweep flow step, blood samples pre and post-membrane were collected, as well as the EtCO_2_ value from the exhalation port of the respiratory membrane.

**Figure 1:**
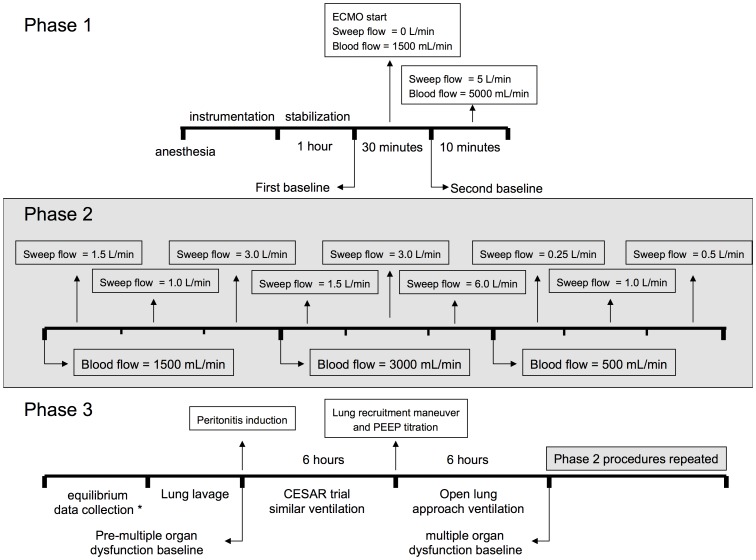
Study procedures timeline. **Phase 1** is the period of anesthesia, instrumentation and stabilization of animals; **Phase 2** is the period of data collection to analyze gas transfers in the normal animals; and **Phase 3** is the phase of equilibrium analysis *, lung lavage, peritonitis induction, and multiple organ failure installation. At the end of phase 3 the data collection described in the phase 2 was repeated, now in the animals with multiple organ failure installed. Gray grounds show the periods of data collection to the current manuscript. *The equilibrium data collection and analysis are the focus of other manuscript.

After this first sequence of data collection, we induced lung injury through surfactant depletion (lung lavage with aliquots of 1 liter of normal saline at 37°Celsius until the P/F ratio was <50) and we induced sepsis due to fecal peritonitis. The peritonitis was induced through the injection of 1 g/kg of feces into the peritoneal cavity. Antimicrobial treatment with 15 mg/kg of amikacin and 500 mg of metronidazol was administered four hours after the peritonitis induction. This interval was used because in previous experiments this period was associated with higher plasmatic interleukin-6 concentrations. [13] Amikacin and metronidazole were used due to the adequacy in peritoneal-fecal septic shock in previous studies. [13], [15] The PEEP used was titrated after a stepwise alveolar recruitment maneuver, in steps of 2 cmH_2_O down from 25 cmH_2_O, looking for the best dynamic compliance. The PEEP was set 2 cmH_2_O higher than the PEEP corresponding to the best compliance. After the induction of peritonitis, a period of twelve hours was awaited for the MOF installation, based on previous experiments.[12]; [15] Hallmarks of MOF in the model are persistent hypotension (ABPm<65 mmHg for 30 minutes), metabolic, renal, and respiratory dysfunctions. Table S1 shows the characterization of the organs failure during the twelve hours evolution and immediately before the second sequence of data collection. After MOF installation, the same sequence of data collection described above was repeated (except for the baseline with and without gas flow). The timeline of the study is shown in the figure 1.

Classically CO_2_ transfer is measured by computing the CO_2_ exhaled from the ECMO device. This approach avoids measuring blood CO_2_ transfer through the trans-membrane CO_2_ gradient, which can suffer from inaccuracies in the calculation of blood CO_2_ contents, especially with major pH and pCO_2_ variations during the passage of blood through the respiratory membrane. [6] To explore these two different methods, we measured the correlation and agreement of both techniques. For all other analyses, we used the gold standard CO_2_ transfer computed using the CO_2_ exhaled from the respiratory membrane.

### Statistical Analysis

Data were predominantly non-normal as assessed by the Shapiro-Wilk goodness-of-fit model and are shown as median and percentiles 25^th^ and 75^th^. Non-paired data were analyzed with the Mann-Whitney’s or Kruskal-Wallis` tests as appropriate; paired data were analyzed with Wilcoxon`s and Friedman’s test. *Post-hoc* analyses were performed using the Tukey`s “Honestly Significantly Different” (HSD) test. Correlation and agreement analyses were done between the CO_2_ transfer measured through the blood CO_2_ gradient pre- and post-membrane and the CO_2_ transfer measured through the CO_2_ exhaled from the respiratory membrane with the correlation scatter plot, the Pearson correlation method, and the Bland – Altman diagram. Multivariate analyses were performed using a backward elimination mixed generalized model with the animal as random factors in order to account for the within-subject correlation among repeated observations. The Markov chain Monte Carlo procedure with 10,000 simulations to reach the equilibrium of distributions was used to retrieve a fixed probability of each resulting independent variable from the mixed generalized model after the backward elimination. Collinearity among the independent variables was tested with the Spearman’s test of correlations in a matrix including all variables tested. Variables with the r coefficient >0.85 were further tested for multicollinearity with the variance inflation factor (VIF). Variables with a VIF<2.5 were considered appropriate to the analyses. The pseudo-R^2^ was calculated for each model in order to show their goodness-of-fit; the calculation was performed with the squared ratio between the Spearman correlation between the fitted values of the model and the original values retrieved from the experiment. The R free source statistical package and comprehensive-R archive network (CRAN)-specific libraries were used to build the graphics and to do all the statistical analyses [19].

## Results

Five animals were studied. Four animals used a 20-French catheter and one animal used a 21-French catheter to drain the blood to the ECMO device. Three animals used 21-French return catheter from the ECMO system, and two used 20-French catheter. Data on the baseline hemodynamics, respiratory variables, and support measures collected at baseline and just before the second sequence of data collection are described in table 1. The total number of lung lavages needed to achieve lung injury was 8, 12, 16, 4, and 6, to reach a PaO_2_ of 54, 50, 50, 46 and 47 mmHg in each animal respectively. The animals were ventilated with a PEEP = 10 cm H_2_O and a FiO_2_ = 1 at this time. The second sequence of data collection, after the induction of multiorgan failure, occurred 1420 [1384,1508] minutes after the first sequence of data collection and 735 [720,740] minutes after the peritonitis induction. Table S1 shows the characterization of MOF at the time of the second sequence of data collection, and at the worst organ function measured within the 735 [720,740] minutes between peritonitis induction and the second sequence of data collection. The fluid balance between the periods of data collection was positive in 8386 [6734,14534] mL of lactated Ringer’s solution. The ACT controls 6, 12, and 18h after the first baseline measurement were 2.0 [2.0,2.6], 2.2 [1.7,2.6] and 1.8 [1.7,2.0] times the control (first baseline) respectively.

**Table 1:** Hemodynamic, respiratory and support measures at baseline (ongoing ECMO blood flow and without sweeper flow) and immediately before MOF induction.

	Initial - baseline	MOF - baseline	P value*
**Hemodynamic** §			
Heart rate – beats/minute	130 [129,135]	170 [138,174]	0.314
ABPm - mmHg	140 [123,146]	62 [54,78]	0.038
PAPm – mmHg	49 [36,50]	44 [34,54]	0.784
CVP – mmHg	7 [5], [8]	16 [10], [19]	0.063
PAOP – mmHg	13 [10], [13]	14 [7], [18]	1.000
CO – L/min	5.9 [5.8,6.3]	4.9 [3.7,5.5]	0.058
RVSW – (mL.mmHg)/beat	27 [19,29]	11 [7], [11]	0.036
LVSW - (mL.mmHg)/beat	78 [71,87]	18 [15,24]	0.026
PVR – dyn. seg^−1^.(cm^5^)^−1^	379 [353,508]	653 [233,757]	0.313
SVR – dyn. seg^−1^.(cm^5^)^−1^	1765 [1310,1871]	647 [553,849]	0.043
**Respiratory** ¶			
Ventilatory mode	Volume controlled	Pressure controlled	
PaO_2_ - mmHg	82 [64,94]	77 [69,93]	0.814
Sat O_2_ - %	94 [88,96]	92 [86,99]	1.000
PaCO_2_ - mmHg	39 [32,40]	42 [35,47]	0.813
FiO_2_	0.30 [0.30,0.40]	0.30 [0.30,0.45]	0.813
Tidal volume - mL	600 [580,625]	180 [145,294]	0.063
Resp. rate –breaths/min	20 [18,30]	15 [10,25]	0.588
P_peak_ – cmH_2_O	31 [28,39]	30 [27,33]	0.313
P_plateau_ – cmH_2_O	17 [17], [22]	30 [27,33]	0.100
PEEP – cmH_2_O	5 [5,5]	18 [18], [20]	0.030
C_st_ – mL/cmH_2_O	50 [7,54]	17 [14,29]	0.053
Pulmonary Shunt - %	20 [11,28]	45 [38,69]	0.036
**Support**			
Norepinephrine –mcg/kg/min	0	0.4 [0.2,1.3]	
ECMO - bloodflow – mL/min	1500	2520 [1750,3690]	
ECMO - sweepflow – L/min	0	8 [5], [12]	

Data were collected just before the beginning of the standardized blood and sweep flow sequences and gas transfer data collection.

* P value of the comparison between pre and after MOF.

§ ABPm denotes mean arterial blood pressure, PAPm – mean pulmonary artery pressure, CVP – central venous pressure, PAOP – pulmonary artery occlusion pressure, CO – cardiac output, RVSW and LVSW – right and left ventricle stroke work respectively and PVR and SVR – pulmonary and systemic vascular resistance respectively.

¶ C_st_ denotes respiratory static compliance.

Figure S1 shows the correlation and agreement between the CO_2_ transfer measured through the CO_2_ exhaled from the respiratory membrane and measured through the trans-membrane gradient of CO_2_. In spite of the good correlation and low bias, the distribution of differences between the upper and lower limits of the Bland-Altman plot precludes the use of one technique instead the other. As already posed, we used the classical CO_2_ exhalation technique.

### Determinants of Oxygen and Carbon Dioxide Transfers

**Figure 2:**
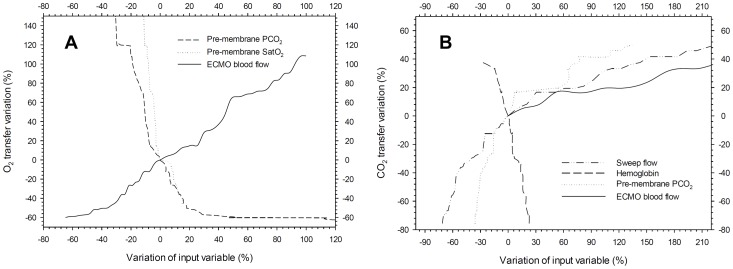
Spider plots showing the strength of association between O_2_ transfer (Panel A) and the variables extracted from the multivariate analysis and the association between CO_2_ transfer (Panel B) and the variables extracted from the multivariate analysis. All data were collected from the animals, with and without multiple organ failure. The input variables are shown in the legend.

**Figure 3:**
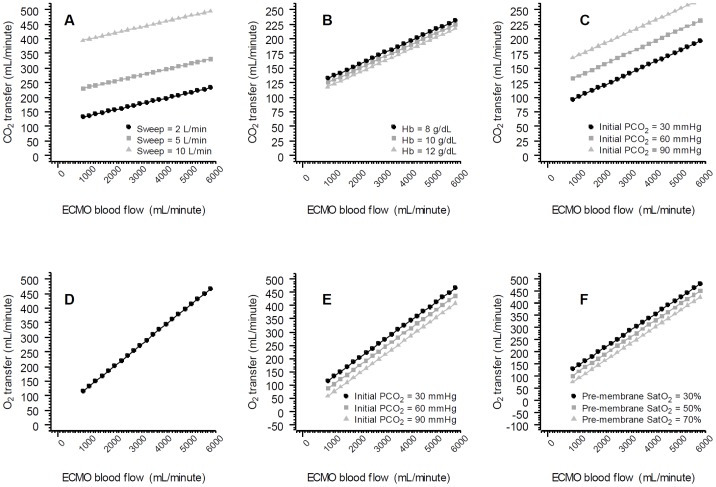
Mathematical marginal model exploring the O_2_ and CO_2_ transfers according to the progressive elevation of ECMO blood flow. The default variables were: sweep flow = 2 L/minute, initial pre-membrane SatO_2_ = 30%, initial pre-membrane PCO_2_ = 30 mmHg and initial hemoglobin = 8.0 g/dL. The marginal model is derived from the multivariate mixed model. **Panel A** shows the CO_2_ transference resultant from sweep flow variation. **Panel B** shows the CO_2_ transference resultant from hemoglobin variation. **Panel C** shows the CO_2_ transference according to the initial PCO_2_ variation. **Panel D** shows the O_2_ transference resultant from ECMO blood flow variation. **Panel E** shows the O_2_ transference resultant from initial PCO_2_ variation. **Panel F** shows the O_2_ transference according to the initial pre-membrane SatO_2_ value.

Two multivariate models were built (Table 2). The first, to assess the association between oxygen transfer as the dependent variable and blood flow, oxygen saturation, PaCO_2_, temperature, base excess, pH, gas flow, and hemoglobin as independent variables. The second, to assess the association between carbon dioxide transfer and blood flow, oxygen saturation, PaCO_2_, temperature, sweep flow, and hemoglobin. In both models, the laboratory data were obtained from the pre-membrane port. In the multivariate analysis, oxygen transfer was associated with ECMO blood flow, pre-membrane SatO_2_, and pre-membrane PaCO_2_. The CO_2_ transfer, on the other hand, was associated with gas flow, pre-membrane PaCO_2_, blood flow and negatively with hemoglobin. The graph correlating the variations in the main variables associated with gas transfers in the multivariate analysis and gas transfers per se are shown in the spider plot presented in the figure 2. The marginal-model plots extracted from the regression model are shown in figure 3, in order to facilitate the understanding of the isolated effect of each variable.

**Table 2:** Backward elimination multivariate analysis exploring variables associated with oxygen and carbon dioxide transfer during ECMO support.

Oxygen transfer analysis			
Variable §	Beta-unstandardized coefficient ^#^	P value	VIF *
**Blood flow (mL/min)**	0.066	<0.001	1.03
**O_2_ saturation (%)**	−1.669	<0.001	1.30
**CO_2_ partial pressure (mmHg)**	−0.964	0.001	1.27
**Carbon dioxide transfer analysis**			
**Variable** §	**Beta-unstandardized coefficient ^#^**	**P value**	**VIF** *
**Blood flow (mL/min)**	0.017	<0.001	1.62
**Sweep (L/min)**	32.910	<0.001	1.62
**CO_2_ partial pressure (mmHg)**	1.192	<0.001	1.07
**Hemoglobin (g/dL)**	−3.478	0.042	1.24

This multivariate analysis was performed using a generalized linear mixed model adjusted to each animal with a backward elimination.

The initial dependent variables in the oxygen transfer analysis were blood flow, O_2_ saturation, PaCO_2_, temperature, base excess, pH, sweep flow and hemoglobin. Base excess and pH were eliminated from the analysis in order to reduce the VIFs bellow the threshold of 2.5, keeping the PaCO_2_ in the model. Sweep flow, temperature and hemoglobin dropped out during the backward elimination of the multivariate analysis. The coefficient of determination of the final model (pseudo - R^2^) was 0.81.

The initial dependent variables in the carbon dioxide transfer analysis were blood flow, O_2_ saturation, PaCO2, temperature, sweep flow and hemoglobin. Temperature and O_2_ saturation dropped out during the backward elimination of the multivariate analysis. The coefficient of determination of the final model (pseudo - R^2^) was 0.89.

§ The blood samples were acquired from the pre-membrane port.

* VIF denotes variance inflation factor.

#Beta-unstandardized coefficient denotes the estimated variation of the oxygen transfer in mL/min for each unit (the units are cited in the table) variation of the independent variables.

### Laboratory Results Pre and Post Membrane

Table 3 shows the laboratory results before and after the passage of blood through the membrane both in health and after the induction of multiple organ failure. We found an increase in the pH in the post-membrane blood, a slight reduction in the ionized calcium value and a rise in the chloride concentration after the passage of blood through the membrane. The elevation in the pH was more accentuated during MOF. The effects of different ECMO settings on the laboratory results are shown in tables 4 and 5. Additionally, these tables explore the effect of multiorgan failure on the laboratory results. The pH variation is of note, mainly in MOF measurements, associated with a higher pre-membrane PCO_2_ and lower pre-membrane oxygen saturation. The higher lactate levels in MOF measurements also deserve attention. The potassium levels were higher during MOF; however the effect of membrane passage on the electrolytes were the same in normal and MOF measurements.

**Table 3:** Comparison of laboratory variables between the blood samples acquired before and after the oxygenation membrane, during the baseline and the MOF clinical conditions.

	Baseline ^&^				Multiorgan dysfunction syndrome ^&^			
	Pre-membrane	After-membrane	Difference	P value *	Pre-membrane	After-membrane	Difference	P value *
**pH**	7.50 [7.46,7.54]	7.60 [7.55,7.65]	0.09 [0.07,0.13] §	<0.001	7.19 [6.92,7.32]	7.41 [7.13,7.5]	0.21 [0.13,0.25]	<0.001
**PaO_2_– mm Hg**	41 [37,49]	422 [341,463]	379 [305,408]	<0.001	38 [28,55]	354 [188,441]	325 [151,406]	<0.001
**PaCO_2_– mm Hg**	35 [32,39]	25 [22,27]	−10 [−14,−7] §	<0.001	59 [47,91]	34 [28,45]	−28 [−50,−14]	<0.001
**SatO_2_ - %**	64 [61,67]	100 [100,100]	35 [32,38] §	<0.001	48 [32,61]	100 [100,100]	52 [39,68]	<0.001
**SBE – mEq/L**	4.7 [3.1,6.0]	4.0 [2.7,5.6]	−0.6 [−1.2,0.1] §	<0.001	−9.4 [−13.1,0.4]	−7.2 [−11.3,5.5]	1.5 [0.5,3.0]	<0.001
**Lactate – mEq/L**	1.8 [1.1,2.0]	1.7 [1.0,2.1]	0.0 [−0.1,0.1]¶	0.530	5.5 [1.9,10.1]	5.3 [1.6,10.1]	−0.2 [−0.7,0.0]	0.003
**Na – mEq/L**	140 [139,141]	140 [138,140]	−1 [−1, 0] ®	0.061	141 [135,148]	139 [34,147]	−1 [−2,−1]	0.240
**K – mEq/L**	3.3 [3.2,3.5]	3.3 [3.2,3.4]	0.0 [−0.1,0.0]	0.115	4.7 [4.2,5.3]	4.6 [4.2,5.3]	0.0 [−0.1,0.0]	0.108
**Ca – mEq/L**	1.28 [1.27,1.32]	1.26 [1.23,1.29]	−0.03 [−0.05,0.00]	<0.001	1.28 [1.19,1.36]	1.24 [1.17,1.33]	−0.03 [−0.05,−0.01]	<0.001
**Cl – mEq/L**	105 [104,107]	107 [105,108]	2 [0,2]	<0.001	101 [97,105]	104 [99,110]	2 [1], [2]	<0.001
**Hemoglobin – g/dL**	11 [10], [12]	11 [10], [13]	0.1 [−0.7,0.6]	0.643	9.0 [8.1,9.3]	8.8 [8.0,9.4]	0.0 [−0.2,0.2]	0.845
**Glucose – mg/dL**	108 [95,121]	110 [96,119]	−1 [−3,3]	0.555	85 [76,91]	85 [78,89]	1 [−2,2]	0.938
**O_2_ transfer – mL/min**	87 [35,148]				90 [44,156]			
**CO_2_ transfer – mL/min**	91 [64,139] §				140 [102,204]			
**Temperature –°Celsius**	37.5 [37.2,38.1] §				38.5 [38.2,38.8]			

* P value of the comparison of variables between pre and after membrane (Wilcoxon U test).

# SBE denotes standard base excess.

&There were 45 measurements analyzed from five animals in each clinical situation (Baseline or multiorgan dysfunction syndrome).

§ P<0.001 vs Multiorgan dysfunction syndrome (Wilcoxon U test).

¶ P = 0.008 vs Multiorgan dysfunction syndrome (Wilcoxon U test).

®P = 0.041 vs Multiorgan dysfunction syndrome (Wilcoxon U test).

The ECMO settings were pooled from the timepoints described in the phase 2 of the figure 1, and were the same in both analyzed conditions (Baseline and MOF).

**Table 4:** Comparison of laboratory variables between the blood samples acquired pre and after the oxygenation membrane, during different blood flows with and without MOF.

		Blood flow = 500 mL/min		Blood flow = 1500 mL/min		Blood flow = 3000 mL/min	
		Non-MOF (n = 15)	MOF (n = 15)	Non-MOF (n = 15)	MOF (n = 15)	Non-MOF (n = 15)	MOF (n = 15)
**pH**	**Pre-membrane**	7.46 [7.43,7.50]	7.30 [7.04,7.32]	7.51 [7.49,7.53]	7.15 [6.89,7.33]	7.55 [7.52,7.58]	7.13 [6.91,7.35]
	**After-membrane**	7.61 [7.53,7.67]*	7.46 [7.27,7.63]*	7.58 [7.56,7.64]*	7.34 [7.12,7.52]*	7.62 [7.59,7.66]*	7.23 [7.07,7.49]*
	**Difference**	0.14 [0.11,0.20]^#&^	0.25 [0.22,0.35]	0.08 [0.07,0.12]^#^	0.20 [0.15,0.24]	0.07 [0.05,0.09]^#^	0.13 [0.12,0.18]
**PaO_2_– mm Hg**	**Pre-membrane**	38 [37,48]	33 [28,48]	42 [39,62]	41 [33,59]	43 [35,44]	40 [34,56]
	**After-membrane**	442 [417,500]*	459 [367,479]*	431 [295,466]*	384 [295,420]*	376 [257,398]*	262 [187,334]*
	**Difference**	408 [378,452]	429 [322,452]	381 [233,410]	354 [258,381]	332 [222,352]^#^	232 [145,294]
**PaCO_2_– mm Hg**	**Pre-membrane**	40 [35,44]	57 [52,84]	35 [36,39]	73 [51,95]	32 [29,34]	69 [44,100]
	**After-membrane**	23 [20,27]*	26 [22,31]*	25 [22,29]*	40 [31,46]*	25 [22,27]*	45 [32,56]*
	**Difference**	−16 [−18,−13]^#&^	−29 [−52,−25]	−10 [−11,−9]^#^	−30 [−50, −21]	−7 [−9,−6]^#^	−20 [−46,−13]
**SatO_2_ - %**	**Pre-membrane**	62 [60,64]	41 [32,44]	66 [65,67]	55 [39,64]	63 [61,73]	56 [41,63]
	**After-membrane**	100 [99,100]*	100 [99,100]*	100 [99,100]*	100 [99,100]*	100 [99,100]	100 [99,100]*
	**Difference**	38 [36,40]^#^	59 [56,68]	34 [32,35]^#^ §	45 [36,60]	37 [27,39]^#^	44 [37,59]
**SBE – mEq/L**	**Pre-membrane**	4.2 [3.2,5.2]	−9.4 [−10.9,−1.9]	5 [3.0,6.1]	−8.1 [−14.5,0.9]	4.7 [3.7,7.4]	−9.3 [−13.5,−1.9]
	**After-membrane**	3.7 [2.5,4.8]	−5.5 [−10.2, 6.4]	4.3 [1.5,5.7]	−5.1 [−12.3,2.9]	4.2 [3.4,6.1]	−7.2 [−11.8, −1.1]^#^ *
	**Difference**	−0.6 [−1.2, −0.2]^#^	1.6 [0.8,5.8]	−0.6 [−1.0,0.2]^#^	1.7 [0.8,3.0]	−0.5 [−1.3, −0.3]^#^	1.0 [0.0,2.1]
**Lactate – mEq/L**	**Pre-membrane**	1.8 [0.9,2.1]	5.5 [2.8,9.8]	1.6 [1.1,1.8]	5.5 [2], [11]	2.0 [1.8,2.4]	5.0 [1.0,10.3]
	**After-membrane**	2.0 [1.0,2.0]*	5.5 [2.0,9.8]	2.0 [1.0,2.0]*	5.0 [2.0,10.3]	2.0 [1.0,2.0]*	4.5 [1.0,10.3]
	**Difference**	0.1 [0.0,0.2]^ #^	0.0 [0.0,0.0]	0.2 [0.0,0.4]^#^	−0.5 [−1.0,0.0]	−0.1 [−0.6,0.0]	0.0 [−1.0,0.0]
**Na – mEq/L**	**Pre-membrane**	141 [139,142]	140 [135,148]	141 [139,141]	141 [135,148]	140 [138,141]	141 [135,147]
	**After-membrane**	140 [139,141]*	138 [134,146]*	139 [138,140]*	139 [134,147]*	139 [138,140]	139 [134,146]*
	**Difference**	−1 [−2,−1]^#^	−2 [−2,−1]	−1 [−2,−1]	−1 [−1,−1]	0 [−1,0]	−1 [−1,0]
**K – mEq/L**	**Pre-membrane**	3.3 [3.2,3.4]	4.5 [4.0,5.3]	3.3 [3.3,3.4]	4.5 [4.0,5.3]	3.4 [3.3,3.5]	4.5 [4.0,5.3]
	**After-membrane**	3.0 [3.0,3.0]	4.5 [4.0,5.3]	3.0 [3.0,3.0]	4.5 [4.0,5.3]	3.0 [3.0,3.0]*	4.5 [4.0,5.3]
	**Difference**	−0.2 [−0.3,0.0]	0.0 [0.0,0.0]	−0.3 [−0.3, −0.1]	0.0 [0.0,0.0]	−0.4 [−0.5, −0.3]^#^	0.0 [0.0,0.0]
**Ca – mEq/L**	**Pre-membrane**	1.29 [1.27,1.31]	1.27 [1.19,1.34]	1.28 [1.26,1.31]	1.28 [1.23,1.34]	1.31 [1.28,1.33]	1.31 [1.22,1.37]
	**After-membrane**	1.24 [1.22,1.28]*	1.21 [1.17,1.30]	1.26 [1.23,1.29]	1.23 [1.20,1.30]*	1.27 [1.26,1.29]*	1.28 [1.20,1.35]*
	**Difference**	−0.04 [−0.05, −0.02]	−0.05 [−0.05, −0.02]	−0.01 [−0.04,0.02]	−0.03 [−0.04, −0.01]	−0.03 [−0.04,0.00]	−0.02 [−0.03, −0.01]
**Cl – mEq/L**	**Pre-membrane**	105 [104,108]	100 [97,104]	106 [106,108]	101 [98,106]	104 [104,105]	101 [98,104]
	**After-membrane**	107 [107,110]	102 [99,107]*	106 [106,108]	104 [99,108]*	106 [105,108]*	104 [100,110]*
	**Difference**	2 [2], [3]	2 [1], [2]	1 [0,1]^ #^	2 [1], [2]	2 [1], [3]	2 [1], [2]
**Hemoglobin** **– g/dL**	**Pre-membrane**	10.7 [10.1,11.7]	8.7 [7.7,9.1]	11.0 [10.5,12.4]	9.1 [8.7,9.3]	11.0 [10.5,12.5]	9.1 [8.2,9.7]
	**After-membrane**	10.8 [10.0,11.6]	8.6 [8.2,8.9]	11.2 [10.9,13.5]	9.0 [7.9,9.5]	11.2 [10.0,13.0]	9.1 [8.0,9.5]
	**Difference**	−0.2 [−0.6,0.2]	0.0 [−0.2,0.6]	0.3 [−0.7,0.9]	0.0 [−0.2,0.1]	0.2 [0.1,0.3]	−0.1 [−0.3,0.1]
**Glucose – mg/dL**	**Pre-membrane**	110 [91,114]	82 [75,91]	107 [96,119]	86 [75,89]	109 [104,123]	87 [77,92]
	**After-membrane**	110 [91,114]	85 [77,89]	108 [98,123]	84 [76,86]	111 [105,121]	86 [79,89]
	**Difference**	−1 [−3,1]	2 [0,2]	−1 [−5,4]	1 [−2,2]	0 [−2,3]	−1 [−3,1]
**O_2_ transfer** **– mL/min**		34 [31,35]^#&^	42 [40,45]^&^	87 [77,107]	94 [72,144]	185 [146,203]	180 [138,271]
**CO_2_ transfer** **– mL/min**		61 [33,84]^#&^	78 [65,109]^&^	91 [66,122]^ #^	148 [129,173]	148 [93,174]^ #^	243 [185,288]
**Temperature** **– °Celsius**		37.5 [37.2,38.1]^#^	38.5 [38.2,38.8]	37.5 [37.2,38.1]^#^	38.5 [38.2,38.8]	37.5 [37.2,38.1]^#^	38.5 [38.2,38.8]

* P<0.05 vs pre-membrane; # P<0.05 vs MOF; SBE denotes standard base excess and n = 15 denotes fifteen measurements from five animals.

&Friedman’s test P<0.017, Tukey`s HSD *post-hoc* analysis P<0.05 vs Blood flow = 1500 and 3000 mL/min.

Friedman’s test P<0.017, Tukey`s HSD *post-hoc* analysis P<0.05 vs Blood flow = 3000 mL/min.

§ Friedman’s test P<0.017, Tukey`s HSD *post-hoc* analysis P<0.05 vs Blood flow = 500 mL/min.

**Table 5:** Comparison of laboratory variables between the blood samples acquired pre and after the oxygenation membrane, during different ratios between sweep and blood flows, with and without MOF.

		Sweep flow<blood flow		Sweep flow = blood flow		Sweep flow>blood flow	
		Non-MOF (n = 15)	MOF (n = 15)	Non-MOF (n = 15)	MOF (n = 15)	Non-MOF (n = 15)	MOF (n = 15)
**pH**	**Pre-membrane**	7.49 [7.46,7.52]	7.19 [6.92,7.31]	7.50 [7.47,7.53]	7.30 [7.00,7.36]	7.52 [7.49,7.55]	7.17 [6.90,7.35]
	**After-membrane**	7.56 [7.53,7.60]*	7.35 [7.10,7.48]*	7.60 [7.57,7.65]*	7.35 [7.12,7.53]*	7.65 [7.61,7.70]*	7.46 [7.20,7.62]*
	**Difference**	0.06 [0.05,0.09]^#&^	0.15 [0.11,0.20] ¶	0.09 [0.09,0.12]^#^	0.17 [0.12,0.22]	0.13 [0.08,0.18]^#^	0.27 [0.21,0.33]
**PaO_2_– mm Hg**	**Pre-membrane**	43 [37,61]	40 [28,55]	40 [38,49]	40 [31,52]	40 [37,45]	37 [28,53]
	**After-membrane**	422 [362,432]*	333 [188,467]*	411 [184,457]*	366 [208,428]*	431 [368,494]*	372 [229,448]*
	**Difference**	363 [323,388]¶	304 [150,434]¶	372 [125,413]	335 [170,388]	398 [328,453]^#^	344 [190,412]
**PaCO_2_– mm Hg**	**Pre-membrane**	35 [33,39]	57 [48,90]	36 [34,40]	70 [48,90]	33 [31,37]	67 [47,91]
	**After-membrane**	27 [26,31]*	44 [31,60]*	27 [23,28]*	34 [30,45]*	22 [19,23]*	27 [23,39]*
	**Difference**	−9 [−10,−6]^#&^	−20 [−37,−12]	−11 [−14,−9]^#^	−34 [−50,−21]	−12 [−17,−10]^#^	−38 [−58, −24]
**SatO_2_ - %**	**Pre-membrane**	64 [61,70]	48 [34,62]	65 [61,67]	48 [35,58]	64 [62,70]	48 [30,61]
	**After-membrane**	100 [99,100]*	100 [99,100]*	100 [99,100]*	100 [99,100]*	100 [99,100]*	100 [99,100]*
	**Difference**	35 [30,38]^#^	53 [38,66]	35 [33,39]^#^ §	52 [43,64]	36 [29,38]^#^	52 [40,69]
**SBE – mEq/L**	**Pre-membrane**	3.9 [2.8,6.0]	−9.7 [−12.3, −2.2]	4.9 [4.3,5.8]	−8.3 [−13.7,0.9]	4.6 [3.7,5.9]	−9.1 [−13.5,0.6]
	**After-membrane**	3.4 [1.4,4.7]*	−7.6 [−11.3,2.4]*	5.0 [2.8,5.7]*	−6.6 [−11.1,3.1]*	4.1 [3.4,6.0]*	−7.2 [−11.5,6.5]
	**Difference**	−0.9 [−1.2, −0.1]^#^	0.7 [0.0,1.8]	−0.6 [−1.3, −0.3]^#^	1.8 [0.7,2.7]	−0.4 [−0.8,0.2]^#^	2.0 [1.4,3.1]
**Lactate – mEq/L**	**Pre-membrane**	1.7 [1.1,2.1]	5.6 [1.9,9.8]	1.8 [0.9,2.0]	5.2 [2.2,10.4]	1.9 [1.1,2.1]	5.2 [2.1,9.9]
	**After-membrane**	1.7 [1.0,2.0]	5.5 [1.6,9.9]	1.6 [0.9,2.1]*	5.1 [1.5,10.3]	1.8 [1.1,2.1]	5.0 [1.6,9.9]
	**Difference**	0.0 [−0.2,0.1]	−0.2 [−0.7,0.2]	0.0 [−0.2,0.1]	−0.3 [−0.8, −0.1]¶	0.1 [−0.1,0.1]	−0.1 [−0.3,0.0]
**Na – mEq/L**	**Pre-membrane**	141 [139,141]	140 [136,148]	140 [139,141]	140 [135,148]	139 [139,141]	141 [134,148]
	**After-membrane**	140 [139,141]	139 [134,147]*	140 [138,140]*	139 [134,146]*	139 [138,140]*	139 [134,146]*
	**Difference**	0 [−1,0 ]¶	−1 [−1,0]	−1 [−1,−1]¶	−1 [−2,−1]	−1 [−1,0]	−1 [−2,−1]
**K – mEq/L**	**Pre-membrane**	3.3 [3.2,3.4]	4.7 [4.1,5.3]	3.4 [3.3,3.5]	4.7 [4.3,5.5]	3.3 [3.3,3.5]	4.6 [4.1,5.4]
	**After-membrane**	3.2 [3.2,3.4]	4.6 [4.1,5.3]	3.3 [3.3,3.5]	4.6 [4.1,5.5]	3.3 [3.3,3.4]	4.5 [4.2,5.4]
	**Difference**	−0.1 [−0.1,0.1]	0.0 [−0.1,0.0]	0.0 [−0.1,0.0]	0.0 [0.0,0.0]	0.0 [−0.1,0.0]	0.0 [−0.1,0.0]
**Ca – mEq/L**	**Pre-membrane**	1.28 [1.26,1.33]	1.28 [1.21,1.36]	1.30 [1.28,1.33]	1.29 [1.22,1.37]	1.28 [1.27,1.31]	1.28 [1.19,1.35]
	**After-membrane**	1.26 [1.24,1.29]	1.25 [1.20,1.33]	1.26 [1.23,1.29]*	1.24 [1.18,1.34]*	1.24 [1.22,1.28]*	1.23 [1.16,1.32]*
	**Difference**	−0.02 [−0.05,0.01]	−0.01 [−0.03,0.01]	−0.03 [−0.04,−0.02]	−0.04 [−0.05,−0.03]	−0.03 [−0.06,−0.01]	−0.03 [−0.05,−0.02]
**Cl – mEq/L**	**Pre-membrane**	105 [104, 108]	101 [98,104]	105 [104,107]	100 [97,104]	106 [104,107]	101 [98,105]
	**After-membrane**	106 [105,108]	103 [100,110]*	107 [105,109]*	104 [99,108]*	107 [106,109]*	102 [100,107]*
	**Difference**	1 [0,2]	1 [1], [2]	1 [1], [2] §	2 [2], [3]	2 [1], [3]	2 [2,2]
**Hemoglobin** **– g/dL**	**Pre-membrane**	10.9 [10.0,12.8]	9.0 [8.0,9.2]	10.8 [10.6,11.6]	9.1 [8.4,9.4]	11.0 [10.5,12.2]	8.9 [8.1,9.6]
	**After-membrane**	11.0 [9.7,13.0]	9.0 [8.3,9.5]	11.0 [10.6,11.9]	9.2 [8.2,10.7]	11.2 [10.6,13.6]	8.6 [7.8,8.8]*
	**Difference**	0.0 [−0.7,0.2]^#^	0.1 [0.0,0.6]	0.2 [−0.5,0.4]	0.0 [−0.2,0.6]	0.3 [−0.2,0.8]	−0.2 [−0.9,0.0 ]
**Glucose – mg/dL**	**Pre-membrane**	107 [94,121]	82 [76,89]	112 [95,121]	89 [79,93]	108 [100,120]	85 [75,88]
	**After-membrane**	108 [99,117]	82 [78,87]	112 [96,120]	86 [79,90]	108 [97,117]	85 [77,88]
	**Difference**	−2 [−5,4]	2 [−2,2]	−1 [−1,2]	0 [−2,2]	−1 [−3,2]	0 [−1,2]
**O_2_ transfer** **– mL/min**		87 [33,155]	98 [45,163]	84 [35,151]	105 [48,160]	95 [35,144]	103 [40,141]
**CO_2_ transfer** **– mL/min**		64 [35,72]¶ ^#^	114 [66,145]^&^	91 [67,130]¶ ^#^	146 [93,188]	139 [107,172]^#^	198 [124,285]
**Temperature** **–°Celsius**		37.5 [37.2,38.1]^#^	38.5 [38.2,38.8]	37.5 [37.2,38.1]^#^	38.5 [38.2,38.8]	37.5 [37.2,38.1]^#^	38.5 [38.2,38.8]

* P<0.05 vs pre-membrane; # P<0.05 vs MOF; SBE denotes standard base excess and n = 15 denotes fifteen measurements from five animals.

&Friedman’s test P<0.017, Tukey`s HSD *post-hoc* analysis P<0.05 vs Blood flow = 1500 and 3000 mL/min.

¶ Friedman’s test P<0.017, Tukey`s HSD *post-hoc* analysis P<0.05 vs Blood flow = 3000 mL/min.

§ Friedman’s test P<0.017, Tukey`s HSD *post-hoc* analysis P<0.05 vs Blood flow = 500 mL/min.

## Discussion

Our main findings were 1) both oxygen and carbon dioxide transfers were significantly determined by blood flow; 2) the oxygen transfer was also affected by the pre-membrane oxygen saturation, pre-membrane CO_2_ and the carbon dioxide transfer, by the gas flow, hemoglobin level, and the pre-membrane CO_2_; 3) there were no concerning electrolyte disturbances associated with the passage of blood through the ECMO system. The slight electrolyte modifications seen are not sufficient to trigger specific interventions at the bedside, suggesting statistical but not clinical significance.

### The Animal Model

This model was created in order to keep many of the multiorgan dysfunction characteristics seen in intensive care units at the bedside. The cardiovascular, renal, and respiratory functions were moderately or severely impaired (Table S1).

The respiratory static compliance reduction could be partially explained by the PEEP elevation leading to overdistention. However, besides the fall in the static compliance, we also observed a dramatic decrease in oxygenation (P/F ratio of 50 [47,51] mmHg), caused by the elevation of the pulmonary shunt to 45 [38,69]% at the MOF baseline, indicating that we obtained a significant amount of lung collapse. Low compliance associated with lung collapse is an important part of the characterization of ARDS (the baby lung) even if, alike in patients, the component related to the disease severity cannot be separated from the application of higher PEEP levels.

### Oxygenation and Carbon Dioxide Removal

In the multivariate analysis (Table 2), the oxygen transfer was associated with ECMO blood flow, pre-membrane SatO_2_, and pre-membrane PaCO_2_. The association of O_2_ transfer with blood flow is in line with the current understanding of the gas exchange during ECMO. [7] The lower pre-membrane oxygen content together with the maximized post-membrane oxygen content would explain the higher O_2_ transfers with lower pre-membrane SatO_2_. On the other hand, our finding of higher O_2_ transfers attained at lower pre-membrane PaCO_2_ values cannot be ascribed to an increase in the availability of oxygen binding sites in the pre-membrane blood, because low PaCO_2_ values are associated with higher hemoglobin affinity for oxygen and a higher pH. [20] Indeed, we were unable to bring forth a physiological explanation for that finding. In a speculative way, once the low PaCO_2_ is associated with a higher oxygen-hemoglobin affinity, a low pre-membrane PaCO_2_ offers a higher contact surface between blood and the lung membrane in an environment with a higher oxygen-hemoglobin affinity, resulting in a higher oxygen transfer. We cannot exclude, however, that there was some residual confounding between ECMO blood flow and PaCO_2_ even after the multivariate adjustment, considering that lower values of pre-membrane PaCO_2_ were associated with higher ECMO blood flows (Table 3).

The CO_2_ transfer was associated with gas flow, pre-membrane PaCO_2_, blood flow and negatively with hemoglobin (Table 5). The strong association between CO_2_ transfer and gas flow is well described and frequently used at the bedside. [7] We also found an association previously described by Kolobow et al in silicone rubber membranes, [6] between high pre-membrane PaCO_2_ values and high CO_2_ transfers. Of note, the increase in the CO_2_ transfer caused by a 10 mmHg increase in the PaCO_2_ was clinically significant and comparable to an increase by 0.5 L/min in the flow of gas. This finding could be of value at the bedside supporting the use of permissive hypercapnia to help optimize the ECMO CO_2_ removal. Another interesting finding was the positive correlation between blood flow and CO_2_ transfer. The effect of blood flow on the CO_2_ transfer was roughly 1.9 times that of the gas flow (Table 2), suggesting, for example, that the pumpless (arteriovenous) systems may have lower efficiency not only in terms of oxygenation, but also regarding CO_2_ removal. The negative association between the CO_2_ transfer and hemoglobin can possibly be explained by a higher CO_2_ content in the form of bicarbonate in the presence of low hemoglobin levels, which could facilitate in an acid pH in the formation of CO_2,_ which is easily eliminated through the membrane. In this scenario, the Bohr effect promoting CO_2_ liberation from the hemoglobin during the membrane oxygenation is probably of reduced significance.

In agreement with the preceding multivariate analyses, when the data were stratified among the three blood flows used, the O_2_ transfer increased six times from the blood flow = 0.5 L/min to blood flow = 3.0 L/min, and the CO_2_ transfer increased two and a half times (Table 4). The elevation of the gas flow from 0.5 to 2 times the blood flow caused a significant augmentation in the pre-post membrane gradient of CO_2_, consequently increasing the CO_2_ transfer. In contrast, the O_2_ transmembrane gradient and the O_2_ transfer were the same in all sweep/blood flow ratios, suggesting no effect of the gas flow on the oxygenation within the range studied (Table 5). The oxygen transfer, with the ECMO blood flow set in 500 mL/min (Table 4), was lower in the pre-multiorgan failure measurements than in the multiorgan dysfunction phase. This finding could be explained by the strikingly low pre-membrane oxygen saturation, associated with a relatively low pre-membrane PCO_2_ during MOF. The multiorgan failure CO_2_ transfers were consistently higher in the multiorgan measurements than in the normal measurements (Table 4 and Table 5), possibly due to higher pre-membrane PCO_2_ in the MOF measurements.

The pre-membrane PCO_2_ and oxygen saturation are cardiac output- dependent variables. In this way, one can conclude that the cardiac output is a potential gas transfer modulator in patients ECMO supported. This fact deserves more investigation.

### Laboratory Results Pre and Post Membrane

During the passage of blood through the oxygenation membrane, there was an increase in pH, a slight reduction in the ionized calcium, and an increase in the chloride, more accentuated after the induction of the MOF (Table 3; Table 4 and table 5). The Gibbs-Donnan effect,[10]; [12] secondary to the ionization of proteins due to the pH variation could explain, at least in part, the chloride and calcium variations through intracellular or extra-vascular shifts. [21] Although statistically significant, the slight reduction in the calcium concentration was not clinically relevant, different from the previously described hemodynamic impairment associated with hypocalcemia in ECMO supported children. [11] Likewise, the changes in chloride concentrations were of minor clinical significance. These findings diminish the hypothesis that the ECMO-induced modifications of the pH [7] could cause significant electrolytes shifts between the intra and extra-cellular media.[9]; [10].

### Limitations

This study has several limitations: 1) higher O_2_ transfer in the ECMO system does not necessary mean higher oxygen availability to the tissues; [22] 2) the low number of animals used could lead to type II errors, attenuating the validity of our findings of lack of associations. Of note, such limitation would not affect our positive findings; 3) in this manuscript; we focused only on the gas transfer across the respiratory membrane. The analysis of the interaction between ECMO blood flow and cardiac output at different natural lung shunt will be explored elsewhere.

### Conclusions

In summary, we confirmed the common knowledge that blood and gas flows affect oxygen transfer and carbon dioxide removal. We also found that the oxygen transfer was negatively associated with the pre-membrane oxygen saturation and carbon dioxide partial pressure. The carbon dioxide transfer was positively associated with the pre-membrane carbon dioxide partial pressure and blood flow, and negatively associated with the hemoglobin level. There was a clinically significant transmembrane elevation of chloride, and pH, however only the former was associated with blood and gas flow variations.

## Supporting Information

Figure S1 Correlation and agreement between the CO_2_ transfer in the lung membrane, measured through the CO_2_ exhaled from the membrane and through the blood CO_2_ content fall during membrane passage. **Panel A** shows the correlation and **Panel B** shows the agreement.
(TIFF)

Table S1 Characterization of multiorgan dysfunction.
(DOC)

Text S1 Online supplement – Calculations.
(DOC)
