# Supplementary figures and images for: Determinants of Oxygen and Carbon Dioxide Transfer during Extracorporeal Membrane Oxygenation in an Experimental Model of Multiple Organ Dysfunction Syndrome
Source: PLoS One. 2013 Jan 29;8(1):e54954. doi: 10.1371/journal.pone.0054954 (PMC3558498; doi:10.1371/journal.pone.0054954)

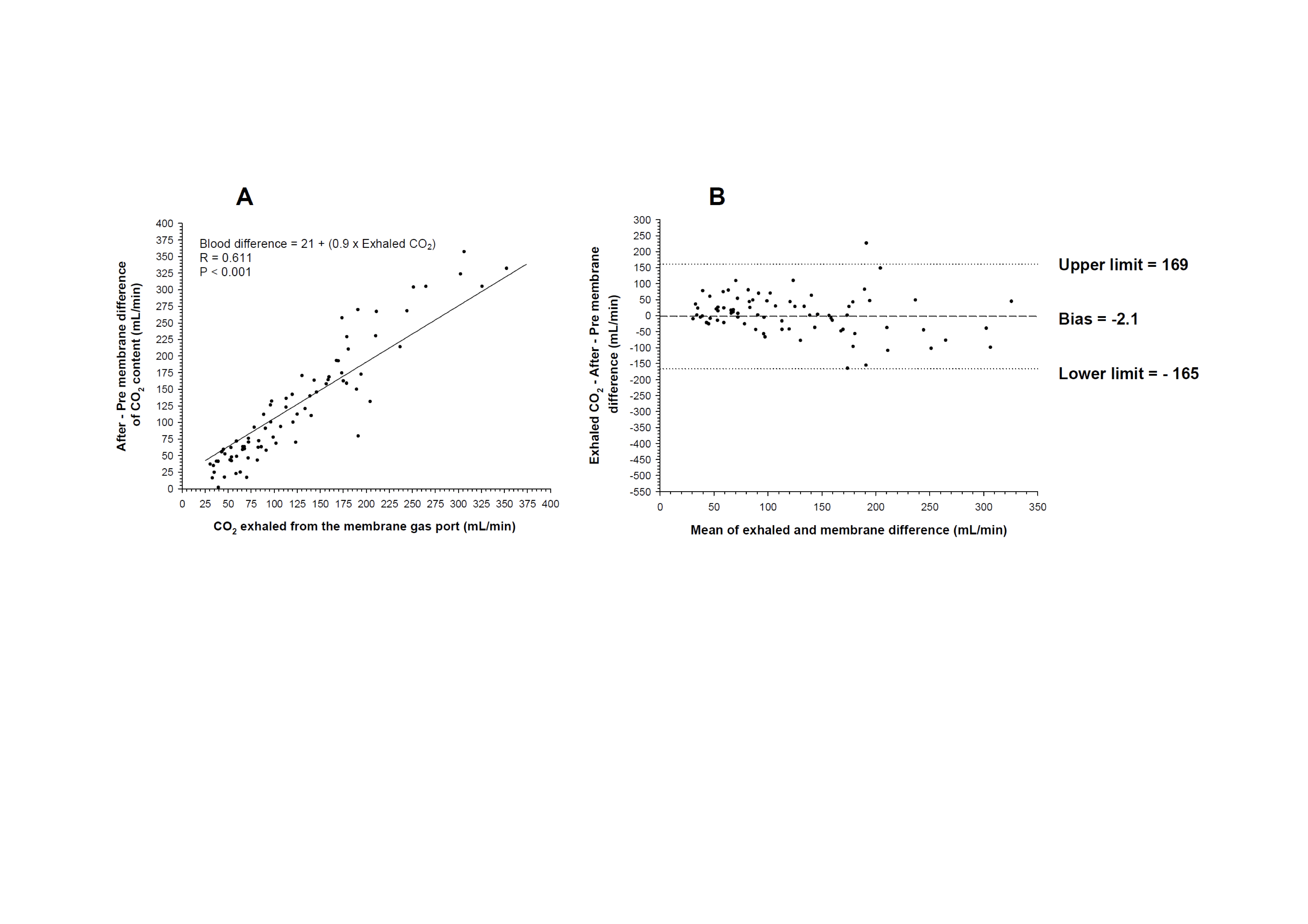

Supplement: Figure S1 — Correlation and agreement between the CO2 transfer in the lung membrane, measured through the CO2 exhaled from the membrane and through the blood CO2 content fall during membrane passage. Panel A shows the correlation and Panel B shows the agreement. (TIFF) [file pone.0054954.s001.tif]
