# Supplementary material for: Determinants of Oxygen and Carbon Dioxide Transfer during Extracorporeal Membrane Oxygenation in an Experimental Model of Multiple Organ Dysfunction Syndrome
Source: PLoS One. 2013 Jan 29;8(1):e54954. doi: 10.1371/journal.pone.0054954 (PMC3558498; doi:10.1371/journal.pone.0054954)
Supplement: Table S1 — Characterization of multiorgan dysfunction. (DOC) [file pone.0054954.s002.doc]

| **Table S1: Characterization of multiorgan dysfunction** | | | | | |
| --- | --- | --- | --- | --- | --- |
|  |  |  | |  | |
| **Cardiovascular function** ^§^ | **Time to the maximum organ dysfunction (hours) ^#^** | | **Maximum organ dysfunction *** | | **Value at the end of 12 hours of observation ^&^** |
| ABPm - mmHg | 7 [6,9] | | 60 [47,98] | | 62 [54,68] |
| CO – L/minute | 9 [4,11] | | 3.9 [3.7,5.5] | | 4.9 [3.7,5.5] |
| Heart rate – beats/minute | 6 [5,10] | | 185 [180,188] | | 170 [138,174] |
| LVSW – mL.mmHg / kg.beat | 7 [7,12] | | 18 [15,21]] | | 18 [15,24] |
| Norepinephrine dosage – mcg/kg/min | 7 [3,9] | | 1.0 [0.6,1.1] | | 0.4 [0.2,1.3] |
| Lactate – mEq/L | 8 [7,10] | | 8.0 [3.0,10.8] | | 7.6 [1.8,10.1] |
|  |  | |  | |  |
| **Renal function** ^¶^ |  | |  | |  |
| SBE – mEq/L | 8 [3,12] | | -8.8 [-9.6,-8.8] | | -8.8 [-8.8,-5.3] |
| Cumulative fluid balance - mL | 12 [11,12] | | 14 [-3,53] | | 14 [-8,53] |
| Urinary flow - mL/kg/h | 9 [6,10] | | 0.13 [0.13,0.27] | | 0.13 [0.13,0.27] |
|  |  | |  | |  |
| **Respiratory function** |  | |  | |  |
| Respiratory static compliance – mL/cmH_2_O | 4 [2,5] | | 9 [5,10] | | 17 [14,29] |
| Pulmonary Shunt - % | 4 [2,7] | | 100 [85,100] | | 45 [38,69] |
|  |  | |  | |  |
|  |  | |  | |  |

# Time between peritonitis induction and maximum organ dysfunction.

* Value of the analyzed variable compatible with the maximum organ dysfunction.

& These values represent the organs dysfunction at the time of second sequence of data collection beginning.

§ ABPm denotes mean arterial blood pressure, CO – cardiac output and LVSW – left ventricle stroke work.

¶ SBE denotes standard base excess.
