# Supplementary material for: Determinants of Oxygen and Carbon Dioxide Transfer during Extracorporeal Membrane Oxygenation in an Experimental Model of Multiple Organ Dysfunction Syndrome
Source: PLoS One. 2013 Jan 29;8(1):e54954. doi: 10.1371/journal.pone.0054954 (PMC3558498; doi:10.1371/journal.pone.0054954)
Supplement: Text S1 — Online supplement – Calculations. (DOC) [file pone.0054954.s003.doc]

**Online supplement**

**Determinants of oxygen and carbon dioxide transfer during extracorporeal membrane oxygenation in an experimental model of multiple organ dysfunction syndrome**

Marcelo Park ^1,2^, Eduardo Leite Vieira Costa ^1,2^, Alexandre Toledo Maciel ^1,2^, Débora Prudêncio e Silva ^2^, Natalia Friedrich ^2^, Edzangela Vasconcelos Santos Barbosa ^2^, Adriana Sayuri Hirota ^2^, Guilherme Schettino ^1^ and Luciano Cesar Pontes Azevedo ^1,2^.

^1^ Research and Education Institute, Hospital Sírio-Libanês, São Paulo, Brazil.

^2^ Intensive Care Unit, Hospital das Clinicas, University of São Paulo School of Medicine, São Paulo, Brazil.

The study was done in the Research and Education Institute of Hospital Sírio Libanês in São Paulo – Brazil.

*Calculations*

The calculations were performed using the following standard formulas:

- Static respiratory compliance [C_st_ – mL/mmHg] = tidal volume / (plateau pressure – PEEP)
- O_2_ transfer [mL/min] = (1.36 x Hb x (After – pre membrane SatO_2_) + 0.0031 x (After – pre membrane PO_2_)) x ECMO blood flow
- CO_2_ transfer [mL/min] = (CO_2_ partial pressure of the exhalation port of the membrane / barometric pressure) x sweep flow in mL/min.
- Blood CO_2_ transfer [mL/min] = ECMO blood flow x ((1 - ((0.0289 x Hb_pre-membrane_) / (3.352 - 0.456 x (SatO_2 pre-membrane_ / 100) x (8.142 - pH_pre-membrane_)))) x 2.226 x 0.0307 + (0.00057 x (37 - Temperature)) + (0.00002 x (37 - Temperature) ^ 2) x PaCO_2 pre-membrane_ x (1 + 10 ^ (pH_pre-membrane_ - 6.086 + (0.042 x (7.4 - pH_pre-membrane_)) + ((38 - Temperature) x 0.00472 + (0.00139 x (7.4 - pH))))) – (1 - ((0.0289 x Hb_after-membrane_) / (3.352 - 0.456 x (SatO_2 after-membrane_ / 100) x (8.142 - pH_after-membrane_)))) x 2.226 x 0.0307 + (0.00057 x (37 - Temperature)) + (0.00002 x (37 - Temperature) ^ 2) x PaCO_2 after-membrane_ x (1 + 10 ^ (pH_after-membrane_ - 6.086 + (0.042 x (7.4 - pH_after-membrane_)) + ((38 - Temperature) x 0.00472 + (0.00139 x (7.4 - pH)))))
- Systemic vascular resistance [SVR – dyn.seg^-1^.(cm^5^)^-1^] = (ABPm – CVP) x 80 / Cardiac output
- Pulmonary vascular resistance [PVR – dyn.seg^-1^.(cm^5^)^-1^] = (PAPm – PAOP) x 80 / cardiac output
- Right ventricle stroke work [RVSW – (mL.mmHg)/beat] = (PAPm – PAOP) x stroke volume x 0.0136
- Left ventricle stroke work [LVSW - (mL.mmHg)/beat] = (ABPm – CVP) x stroke volume x 0.0136
- Standard base excess [SBE – mEq/L] = 0.9287 x (HCO3^-^ - 24.4 + 14.83 x (pH - 7.4))
